# A study on the influence of parents’ educational style of coping with their children’s setbacks experiences on children from grade K3: an exploration based on interviews and qualitative analysis

**DOI:** 10.3389/fpsyg.2026.1732735

**Published:** 2026-04-10

**Authors:** Ziyue Wei

**Affiliations:** Liufang Kindergarten, Huanggang, Hubei, China

**Keywords:** Grade K3 children, parents’s educational style, parent–child relationship, problem response, setback education

## Abstract

This study used interview survey method and grounded theory for qualitative analysis to focus on 100 parents of kindergarten children (aged 5–6 years) in Chinese Mainland, and systematically explore the different ways of parents to deal with children’s setback experiences and their multidimensional effects on children’s psychosocial development. The study identified four core coping styles (negative, indulgent, neglectful, and directive) and revealed their profound effects on children’s self-concept formation, emotional regulation ability, social behavior patterns, prosocial development, and parent–child relationship quality through qualitative analysis. This research has found that directive coping style (especially exploratory guidance type) is most effective in promoting children’s adaptive development, while negative coping style is highly correlated with children’s internalizing problems (such as inferiority complex and anxiety) and externalizing problems (such as aggressive behavior). The research results provide evidence-based scientific guidance for parental education practice and home-school co-education.

## 1 Introduction

Children in the kindergarten senior class (5–6 years old) are currently in the stage of “Initiative VS. Guilt” in Erikson’s theory of psychosocial development. At this stage, children’s self-awareness is significantly enhanced, their psychological theory ability is further developed, and they are able to initially understand their own and others’ thoughts, feelings, and intentions. Their language expression, emotional differentiation (such as being able to distinguish complex emotions such as shame, guilt, pride, etc.), and causal reasoning ability have also rapidly improved. However, children at this stage still have significant self-centered thinking characteristics, and their emotional regulation ability and social problem-solving skills are not yet mature. In the microsocial environment of kindergarten, children inevitably encounter various setbacks: peer conflicts (such as fighting for toys, being excluded), academic challenges (such as difficulty in mastering writing and arithmetic), rule adaptation (such as being misunderstood or criticized), and hindered desires (such as unmet needs). These experiences are essential components of children’s social development. Bandura’s social learning theory emphasizes that children learn coping strategies by observing and imitating the behavior of important others, especially parents. Therefore, how parents respond to their children’s setbacks is not simply an immediate intervention, but a crucial process that deeply shapes their children’s coping patterns, attribution styles, and psychological resilience.

Parents in Chinese Mainland, influenced by traditional Chinese culture and Confucianism, have formed a unique educational style different from that of the West. For example, in some traditional cultural concepts, “filial piety comes from strict stick education” (Shao, 2016), “preference for the first child” (Yan, 2022), and “experiencing setbacks is a good thing” may have a potential profound impact on the educational methods of some parents in Chinese Mainland, especially when dealing with children’s setbacks. Because in some traditional Chinese beliefs, setback education means strict educational methods and styles, and some people who hold traditional beliefs believe that setback education is a good thing. Different Chinese parents have been influenced to varying degrees by traditional culture and have different perspectives on understanding it. However, in Chinese Mainland, there is a lack of detailed research on parents’ coping with their children’s setback experiences, so this paper has unique and novel significance. The purpose of this study is to explore in depth the specific ways of parents in Chinese Mainland to cope with the setback experiences of kindergarten children from Grade K3 and its impact on children’s development. The qualitative research method used in this article, especially grounded theory, provides a unique perspective for revealing the coping mechanisms of parents in the face of children’s setbacks, due to its ability to deeply explore the subjective meaning construction and social interaction process of participants. Through this method, this article is able to gain a more comprehensive understanding of the complex internal logic and true nature behind this phenomenon, laying a solid methodological foundation for exploring emotional support and behavioral response patterns in family education (Chen and Wu, 2023). And the specific research question is: (1) What are the main coping styles exhibited by parents when dealing with their children’s setbacks? (2) What specific impacts do different coping styles have on children’s self-concept, emotional regulation, social behavior, and parent–child relationships? (3) Is there a parental coping model with local characteristics in the context of Chinese culture? By answering the above questions, this study aims to fill the gap in the existing literature on the specific interactive processes of parents from the Chinese mainland in coping with children’s setbacks, and provide evidence-based practical guidance for parent education and home-school cooperation. This article may also provide educational development and reference for Asian countries such as South Korea and Vietnam, which are influenced by similar Confucian traditions, as well as a unique comparative perspective for Western countries with completely different educational styles.

## 2 Literature review

How parents cope with their children’s setbacks is a crucial and enduring issue in the field of child development. However, the traditional Chinese education advocates setbacks education, and believes that it is a good thing for children to experience frustration. The Chinese mainland is affected by the “setback education” in the traditional Chinese culture. Traditional concepts believe that children experiencing setbacks or adversity can exercise their willpower and make them stronger. Under this unique cultural background, although the existing research in The Chinese mainland has indirectly studied this issue from the perspective of parental rearing patterns, there is a lack of in-depth, systematic and targeted research. Existing research generally suggests that parents’ educational methods, as a core component of family socialization practices, profoundly influence children’s emotional, cognitive, and social development. This literature review will sort out relevant literature from three aspects: research on parenting styles and the development of preschool children, research on emotional education for preschool children, and research on setback education for preschool children, in order to establish the theoretical starting point and contribution space of this study.

### 2.1 Research on parenting styles and the development of preschool children

Early research on parental behavior in the world often focused on macro parenting styles. The authoritative, authoritarian, and permissive parenting theoretical frameworks proposed by Baumrind (1971) laid the foundation for understanding the impact of parental behavior on children’s development. Subsequent researchers further deepened this field, such as Maccoby and Martin (1983) expanding parenting styles into two dimensions of responsiveness and demand, forming a more refined classification framework. In recent years, the research on parental rearing patterns and preschool children’s development in The Chinese mainland mainly focuses on: ① the impact of parental rearing patterns on preschool children’s electronic product dependence. For example, inappropriate parenting styles such as excessive parental protection or lack of companionship can lead to children’s screen dependence (Li, 2025), appropriate parenting styles can reduce children’s screen dependence (Liang, 2017), and scientific disciplinary methods can promote children’s rational use of mobile phones (Liu, 2017). ② Research on the impact of parenting styles on sleep quality ofpreschool children. For example, effective parental companionship may have a positive impact on the sleep quality of young children (Li, 2020), parenting styles may affect the sleep quality of young children (Liu et al., 2014), and parents’s educational intervention may affect the sleep quality of young children (Liu, 2019). ③ Research on the influence of parenting styles on the behavior of preschool children, such as negative parenting styles leading to hyperactive behavior of young children (Zou, 2020), parents’s corporal punishment education leading to aggressive behavior of young children (Jia, 2013), and authoritarian parenting styles leading to social problems of children (Song et al., 2023).

### 2.2 Research on emotional education for preschool children

In recent years, research on emotional education of preschool children in The Chinese mainland mainly focuses on: ① research on the impact of family environment on preschool children, for example, parents’ educational intervention can help reduce children’s emotional problems (Deng, 2020), authoritative parenting style has a positive impact on preschool children’s emotional regulation ability (Ding and Liu, 2025), and democratic parenting style can help children control their emotions (Pei, 2024). ② Research on the emotional impact of exercise on preschool children, such as appropriate exercise can alleviate emotional problems of young children (Zhang, 2024), exercise intervention may promote emotional improvement of young children (Chen Lanzhi et al., 2024), and accumulating exercise experience may promote children’s emotional management (Zengfei, 2024). In addition, international research on emotional education also provides important references for this field. For example, Denham’s (1998) study on the development of emotional abilities emphasizes that parents, as key agents of emotional socialization, have a direct impact on the development of children’s emotional understanding and regulation abilities through parents’s response to children’s emotions. On this basis, domestic scholars have also begun to pay attention to the construction of emotional education courses in kindergartens (Yang, 2024), but there is still a lack of systematic research on how parents conduct emotional education in family situations.

### 2.3 Research on setback education for preschool children

In recent years, research on preschool children’s setback education in Chinese Mainland has mainly focused on: ① Methods of preschool children’s setback education, such as providing children with opportunities to fully experience setbacks (Lei, 2024), training children’s frustration tolerance (Ning, 2023), and developing children’s ability to cope with setbacks by using situational games (Lin, 2018). ② The significance of educating preschool children with setbacks, such as promoting their future success (Han, 2013), cultivating their courage to overcome difficulties (Wang, 2012), and developing their ability to independently solve problems (Tang, 2021). However, these studies mostly remain at the level of educational recommendations, with insufficient exploration of parents’ coping behaviors in specific setbacks and their deep impact mechanisms on children’s development.

Although existing research has achieved fruitful results, there are still some gaps. Firstly, the study of parenting styles originated from the Western educational theory but the unique concept of “setback education,” family structure, and value orientation in Chinese culture may shape unique parental coping behaviors (such as the “informed negation type” found in this study), and its influencing mechanism needs to be further explored in the local context. Under the long-term influence of the traditional Confucian culture, the educational creed of “filial piety comes from the stick” (Song, 2017) and the evaluation orientation of “face—saving culture” on children’s behavior in the Chinese mainland (Li, 2020) may make parents’ ways of coping with children’s setbacks show characteristics different from those of Western culture. Secondly, existing studies often use questionnaire survey methods, which lack in-depth characterization of the specific interactive processes, decision-making considerations, and immediate reactions of parents in dealing with setbacks. Qualitative research methods, such as grounded theory, are particularly suitable for revealing the underlying mechanisms by which parents cope with the complex phenomenon of children’s setbacks, as they can delve into the subjective meaning construction and interactive processes of participants (Chen and Wu, 2023). Finally, there is a lack of detailed qualitative comparison regarding the differences within the positive approach of “guidance,” such as the advantages and disadvantages of “experiential” and “exploratory” approaches. Therefore, this study adopts in-depth interviews and grounded theory methods to systematically depict the complex picture of parents from the Chinese mainland coping with setbacks in kindergarten senior children. It not only verifies classical theories, but also aims to identify culturally specific coping styles (such as informed negation/indulgence) through data-rooted method in real situations, and deeply reveal the superiority and underlying mechanisms of “exploratory guidance” compared to other methods, in order to provide more targeted and evidence-based practical guidance for parents’s education and home-school cooperation.

## 3 Research methods: in-depth interviews and systematic qualitative analysis

1) *Research design*: Adopting a phenomenological approach in qualitative research, this study captures parents’ real experiences, specific behaviors, and underlying belief systems in coping with setbacks through in-depth interviews. Grounded theory methods are used for data analysis and theoretical construction. This study conducted data collection from January 2025 to June 2025.
2) *Research subjects*: 100 primary caregivers (parents) of kindergarten children aged 5–6.
3) *Sampling method*: A strategy combining purposive sampling and maximum variation sampling is adopted. The parents participating in this interview come from diverse geographical regions (eastern, central, and western), different types of cities (first-tier, second-tier, third-tier and below), and various family socioeconomic backgrounds in the Chinese mainland, in order to ensure the diversity and representativeness of the sample.
4) *Inclusion criteria*: Children’s age is strictly limited to 5–6 years old; Children and parents have reported normal intellectual development and no known diagnosis of mental or neurological disorders; Parents are the primary caregivers and willing to participate in in-depth interviews.

### 3.1 Data collection

1) *Tool*: Use a semi-structured in-depth interview outline. The core of the outline focuses on asking parents to describe in detail 1–2 typical setbacks that their children have experienced recently (such as specific situations, children’s emotional and behavioral reactions), as well as their own specific reactions throughout the process (including language, behavior, emotions), decision-making process, and post reflection. Open question design encourages respondents to fully describe details and feelings (such as: “How did your child tell you at that time? What was your first reaction? What did you do specifically next? Why did you choose to do it this way? How did you feel with your child afterwards?”).
2) *Process*: The interview is conducted by trained researchers in a one-on-one format in a quiet and private environment (with the option to choose offline or online video). The average interview duration is about 40 min. All interviews were recorded with informed consent and subsequently transcribed verbatim into text.

### 3.2 Data analysis

This article uses a qualitative analysis framework and primarily employs a three-level coding program based on grounded theory:

1) Open coding: Carefully read the transcript of the interview line by line and paragraph by paragraph, conduct preliminary conceptualization and tagging, capture all original information points related to “setback events,” “parental coping behaviors,” “child reactions,” “perceived impact,” etc. (such as “directly blaming the child,” “criticizing even after watching the surveillance,” “discussing solutions together,” “the child becoming afraid to speak,” etc.), and form a large amount of initial code.
2) Axial coding: Classify, compare, and correlate the initial code generated in open coding. Identify the logical relationships between codes (such as causal relationships, conditional relationships, strategic relationships, etc.), and aggregate relevant codes into more general categories or themes. At this stage, the four core parenting styles of “negative,” “overindulgent,” “neglectful,” and “directive” and their subcategories (such as direct blame, understanding before blaming, etc.) are identified, and their relationship with specific child influences (such as internalizing and externalizing problems) is preliminarily correlated.
3) Selective coding: Based on the axial coding, the system integrates all categories and constructs a core category with inherent consistency and explanatory power around the core phenomenon of the study (the impact of parental coping styles on children) – “Parental setback coping style as a key regulator of children’s adaptive development.” Extract the storyline that runs through the data, and continuously compare and revise new data with existing categories and relationships through continuous comparison until theoretical saturation is reached, where newly collected data can no longer generate new categories or relationships.

### 3.3 Reliability and validity guarantee

1) Peer debriefing: During the analysis process, invite another researcher to independently encode a portion of the transcribed text, compare the encoding results, and discuss consensus. The coding content of the author and another researcher in this article is almost identical, with a high degree of consistency, indicating that this article has a very high level of reliability.
2) Members’s checking: Provide preliminary analysis results or key interpretations to some respondents to confirm whether they accurately reflect their experiences and intentions. The participants all expressed that the results were consistent with their own experiences, which reflects good validity.
3) Rich citation of raw data: When presenting research results, a large number of quotes from interviews are used as evidence to enhance the traceability and credibility of the analysis.
4) Reflective recording: Researchers continuously document their thoughts, assumptions, emotional responses, and their potential impact on the research during interviews and analysis, enhancing the transparency of the study. For example, researchers realize that their background as educators may tend to identify with exploratory coping styles, so they pay special attention to equal scrutiny of various coping styles in the coding and analysis process, avoiding preconceived judgments.
5) Research findings: Multidimensional classification and profound impact.

Through systematic coding and analysis of transcribed texts from 100 in-depth interviews, the study clearly identified four dominant styles and subtypes of parents’ handling of children’s setbacks (As the Table 1 shown below), and revealed the complex and diverse psychological and social adaptation outcomes exhibited by children under each style.

**Table 1:** The classification of four core coping styles.

Four dominant styles	Subtypes	Core features
Negative coping style	Direct negation type	Blame the child without understanding the facts
	Informed negation type	Blame the child even after understanding the facts
Indulgent coping style	Direct indulgence type	Unconditional attribution to external factors
	Informed indulgence type	Attributing external factors even after understanding the facts
Neglecting coping style	Directly Neglected Type	Explicitly refuse to listen
	Perfunctorily Neglected type	Lack of focus and emotional investment in response
Guided coping style	Experiential guidance type	Provide solutions based on personal experience
	Exploratory guidance type	Guide children to explore solutions on their own

## 4 Core classification and psychological mechanism of coping styles

### 4.1 Negative coping style

The core characteristic is to attribute setbacks or problems to the child’s own shortcomings or mistakes, often accompanied by criticism, belittlement, and even corporal punishment. Its psychological mechanism may stem from the authoritarian personality of parents, misunderstandings of “strength”, personal growth experiences, or high levels of performance anxiety. Some parents are also influenced by certain traditional beliefs, believing that criticism and adversity can cultivate children’s willpower and make them stronger. This style can be subdivided into:

1. Direct negation type

Parents quickly attribute responsibility to their children without fully understanding the facts. For example:

My son Xiaoming cried and said that his classmates were grabbing his eraser and pushing him, and I immediately became angry: You must have provoked them first! Otherwise, why would they bully you? You only know how to cry, you are useless!’ “(M32, father).

This behavior deprives children of the opportunity to explain and express their feelings, conveying the message that ‘your feelings and defenses are not important, the mistake lies with you’.

2. Informed negation type

Parents will conduct a certain degree of investigation (such as asking teachers, watching surveillance), but regardless of the truth, they will ultimately point the responsibility to the child, emphasizing their personality or ability deficiencies (such as “weak,” “stupid,” “not sensible”). For example, some parents claim that their children have been bullied, but as parents, after watching the security footage, they still accuse their children of “why do not they know how to fight back? So cowardly. “This is more covert than direct blame, and may cause children to feel deeper confusion and injustice (“Although I am a victim, why is it still my fault?”), and internalize negative labels imposed by parents (such as “I am really weak”), forming a negative self-schema.

### 4.2 Indulgent treatment style

The core characteristic is that parents unconditionally attribute setbacks or problems to external factors (others, environment), excessively protect their children from any negative evaluations or consequences, and lack necessary responsibility guidance. Its psychological mechanism may stem from parents’ separation anxiety, compensatory psychology (compensating for their own childhood deficiencies), misunderstanding of “love,” or distrust of children’s autonomy. It can be subdivided into:

1. Direct indulgence type

This type of parents, without fully understanding the facts, directly do not believe that their children are at fault and assume that it is the fault of others (classmates, teachers) or the environment (unreasonable rules), and may engage in intense external aggression (such as insulting others). For example:

My baby said the teacher criticized her today, it must be because the teacher is biased! I will go to the principal’s office tomorrow to complain! What kind of bad teacher! “(F25, mother).

This hinders children from learning to objectively view events and take appropriate responsibility.

2. Informed indulgence type

This type of parents will first investigate the situation, but even if evidence suggests that their child has engaged in inappropriate behavior, they will still seek external reasons to excuse their child and completely shift the responsibility onto others or the situation. For example, in an interview, some parents stated that although they knew their son had broken a female classmate’s hairpin because he found it interesting, they still attributed their son’s terrible behavior to the female classmate wearing a “distracting” hairpin. This reinforces the child’s external attribution tendency, weakens their understanding and introspective ability toward the consequences of their own behavior, and hinders the development of moral responsibility.

### 4.3 Neglecting processing method

The core characteristic is that parents show emotional or behavioral indifference, avoidance, or perfunctory attitude toward their children’s setbacks and accompanying emotional needs. The psychological mechanism may stem from parents’ own experiences of emotional neglect, severe parenting pressure, depressive emotions, or insufficient awareness of the importance of children’s emotions. Some parents may also be influenced by traditional beliefs, believing that letting their children face and solve setbacks independently is a good way of education, so they choose to ignore their children’s confession. It can be subdivided into:

1. Directly neglected type

This type of parents explicitly refuse or interrupt their children’s confession. For example:

As soon as the child spoke up, ‘Mom, I encountered a problem in kindergarten today…’, I interrupted him and said, ‘Didn’t you see that I’m busy? Don’t bother me with this small matter!’ “(F38, mother).

This conveys a hurtful message to the child: your pain is not important to me, you are not worthy of attention, and so on.

2. Perfunctorily neglected type

These parents are formally listening, but lack substantial emotional investment, understanding, or action support. The response is often formulaic, absent-minded (such as “hmm,” “got it,” “okay”), or quickly changes the topic. For example, when a child tells of being ridiculed, parents keep their eyes on the phone screen and casually say, “Oh, then don’t pay attention to him.” This emotional dissonance makes the child feel perfunctory and isolated, and their emotions are not truly seen and contained, hindering the formation of secure attachment and the development of emotional recognition and management abilities.

### 4.4 Guided coping style

The core feature is that parents acknowledge and accept their children’s feelings of frustration, actively guide them to recognize problems, think about solutions, and provide emotional and methodological support in the process. Its psychological mechanism embodies the core characteristics of authoritative parenting: a balance between high responsiveness and high demands. It can be subdivided into:

1. Experiential guidance type

These parents provide solutions directly based on their own experience. For example, in the case of “Alice’s Braids”, in this interview, Alice’s mother mentioned that she heard that Alice was being pulled by mischievous boys on her braids in kindergarten. Alice’s mother learned that such a thing did happen by going to school to watch security footage and talking to teachers. Alice’s mother, Susan, recalled that when she was a little girl in Grade K3, she was also teased by a mischievous little boy who pulled her braid. At that time, Alice’s grandparents advised Alice’s mother Susan to cut off her braid and keep her hair short, as it would be cooler, more convenient, and prevent boys from pulling her braid again.Susan followed this similar experience from her childhood, she also advised her daughter Alice to cut braid, telling Alice that it would be cooler, more convenient, and stop little boys from pulling her hair. Although Alice was initially a bit unhappy about cutting beautiful hair, she ultimately accepted her mother Susan’s suggestion and kept her hair short. And indeed, no little boys pulled her hair afterward. There were indeed no more little boys to pull her hair. In this case, the mother provided specific advice on “cutting short hair” based on similar experiences during her own growth process. This method is efficient and can convey experience, but it may limit children’s opportunities for independent exploration and trial and error learning, and the potential risk is that children overly rely on the “standard answers” provided by their parents.

2. Exploratory guidance type

This type of parents act as guides (rather than decisionmakers), working together with their children to analyze problems, explore multiple possibilities, evaluate the consequences of solutions, and support their children’s choices and implementation. For example, in the case of “Xiaohong’s Pencil”, in this interview, Xiaohong’s parents mentioned that Xiaohong used to bring a very beautiful pencil to the kindergarten classroom every day, and Xiaohong’s deskmate also liked Xiaohong’s pencil very much. Therefore, Xiaohong’s deskmate often secretly took Little Red’s pencil to draw, which made Little Red very unhappy. After Xiaohong returned home from school and told her parents, her parents learned through watching security footage and talking to the teacher that such a thing had indeed happened. So Xiaohong’s parents and Xiaohong discussed together how to solve this matter. Xiaohong’s parents first asked Xiaohong if she had any solutions she had come up with, but Xiaohong shook her head and said she had no idea. So Xiaohong’s father suggested that perhaps the teacher could change Xiaohong’s deskmate, so as to avoid seeing the classmate who made Xiaohong unhappy. Xiaohong’s mother suggested that perhaps Xiaohong can give her deskmate the same pencil, so that Xiaohong and her deskmate can become good friends and avoid the deskmate stealing Xiaohong’s pencil again. Xiaohong’s parents made her think about whether she simply wanted to solve problems or whether she wanted to gain a new good friend while solving problems. After thinking about it, Xiaohong thought her mother’s suggestion was very good, so she brought the same pencil to her deskmate the next day during class. Xiaohong did become good friends with her deskmate and was never stolen by her deskmate again. In this case, the parents supported their child in making a choice and implementing it by asking questions (“What do you think of?”), providing options (changing seats vs. giving pencils), and guiding them to think about the potential outcomes of different solutions (“simply solving problems vs. making friends”). This process is highly consistent with Vygotsky’s zone of proximal development theory, where parents build a bridge between their child’s “current level” and “potential level of development”, effectively promoting the development of their child’s metacognitive, decision-making, and executive abilities.

## 5 Qualitative analysis of the multidimensional impact of parental coping styles on children’s development

Qualitative analysis reveals the profound and multifaceted impact of different parenting styles on children in the kindergarten senior class, which are intertwined and jointly shape the adaptive development trajectory of children.

### 5.1 The influence of negative style

1. Direct negation type

- Negative impacts:

- Self-concept impairment: Frequent accusations lead to children internalizing negative evaluations, resulting in low self-esteem, inferiority complex, and strong self-doubt. For example,the child Xiaojie (from a direct blaming family) shows obvious withdrawal behavior under parents’s observations, when invited to participate in games, he murmurs softly, “I can’t do it, I’ll mess it up.” This is highly consistent with Erikson’s theory of the risk of excessive development of “guilt” at this stage.
- Emotional regulation disorders: Long term critical pressure can easily lead to chronic anxiety, excessive alertness, and even learned helplessness. For example, in this study, it was found that student Xiaoli (from a family of first blaming and then understanding) exhibited a strong fear response (trembling, crying) toward minor criticism (such as the teacher pointing out the color lines in the picture).
- Externalization behavior problem: The imitation mechanism of social learning theory is significantly reflected here. Children who are directly criticized by their parents (especially accompanied by corporal punishment), such as Xiao Ming, are more inclined to imitate this aggressive behavior pattern and impose blame or even violence on their peers (“Deserve it! Stupid!”). At the same time, it is also possible that, like Xiao Gang, due to long-term fear of high-pressure environments, behavior becomes inhibited, overly timid, and withdrawn.
- Destructive parent–child relationship: Children experience that parent–child interaction is dangerous and unsafe (insecure attachment), closing communication channels, leading to relationship alienation and indifference.
- Impaired sense of security: Children lose confidence in their parents’ protection and support, and develop a deep fear of being abandoned (such as worrying about being abandoned by their parents if they do not do well in exams).

B. Possible ‘positive’ impacts (to be interpreted with caution):

Some children may develop extremely high external behavior standards and perfectionism tendencies under high pressure (such as Xiaoming and Xiaogang’s excessive seriousness in their studies). However, this “strictness” often stems from exogenous motivations (such as fear of punishment) rather than endogenous motivations or genuine sense of ability, and its psychological cost is high and unsustainable, not a healthy adaptive manifestation.

2. Informed negation type

The influence of an informed negation type is similar to that of a direct negation type, but children who grow up in an informed negation type may acquire some form of “investigation,” but because its purpose is still to blame, it has not truly cultivated critical thinking for objective analysis.

### 5.2 The influence of indulgent style

1. Direct indulgence type

- Negative impacts

- Self-centeredness and social cognitive bias: Lack of reflection on one’s own inappropriate behavior and understanding of external rules and others’ feelings can easily develop into narcissistic tendencies or a strong sense of power. For example, a child in the case did not receive proper guidance after grabbing the card, it may reinforce his erroneous belief that ‘I can take it if I want’.
- Low resilience to setbacks and emotional instability: When the external environment cannot meet their needs (such as obstructed wishes or restricted rules), they are prone to strong emotional outbursts or unreasonable behavior due to the lack of internal regulatory strategies and the ability to accept “delayed gratification.”
- Social adaptation difficulties: Their self-centered behavior patterns are prone to conflict and exclusion in peer interactions, making it difficult to establish equal and lasting friendships. Long term may face challenges in integrating into collective life.

B. Positive impact

- High self-worth and sense of security: unconditional (but irrational) support from parents allows children to experience a strong sense of love and protection (safety base effect), forming a higher foundation of self-esteem and sense of security. Child Jack(From a family that always attributes mistakes to others) showed boldness and willingness to try in class.
- Extraversion and exploratory willingness: Less worry about negative evaluations, making children more willing to showcase themselves and try new things (such as painting innovation) in social and cognitive activities, and having a certain behavioral activation tendency.
- Intimacy of parent–child relationship: Children usually see their parents as strong allies and are willing to share.

2. Informed indulgence type

The influence of the informed indulgence type is basically similar to that of the direct ndulgence type, but children who grow up in the informed indulgence type may learn a little bit of “investigation” form, but its purpose is to find a scapegoat, strengthen external attribution bias, and do not help develop true empathy and perspective taking ability.

### 5.3 The influence of neglecting style

1. Directly neglected type

- Negative impacts

- Emotional deprivation and attachment issues: Children’s basic emotional needs (being seen, understood, and comforted) continue to be unmet, experiencing profound emotional neglect. This can easily lead to insecure attachment (such as avoidant or chaotic), forming core beliefs in children’s hearts that “I am not worthy of love” and “my feelings are not important”.
- Inhibition of emotional expression and lack of empathy: Children learn that expressing emotions is ineffective or even dangerous, gradually suppressing emotional expression (such as in the case where the child did not respond when seeing others fall), leading to an increased risk of expressive disorders – difficulty in identifying and describing their own and others’ emotions. The normal development of the mirror neuron system may be hindered, leading to poor development of empathy ability.
- Low self-worth and lack of security: Parents’ indifference directly conveys the message of “I am not important” and “I may be abandoned”, seriously damaging children’s sense of self-worth and security.
- Hindered development of social skills: Lack of parental demonstration and guidance, children lack opportunities to learn how to initiate interactions, resolve conflicts, express needs, etc., which may lead to social withdrawal or clumsiness.
- Attention and executive function challenges: Parents’ neglect or refusal may inadvertently “train” their children to handle information in the same way. For example, Xiao Dai, who grew up neglect, showed significant distraction and difficulty in consistently completing tasks in kindergarten activities.

B. Possible ‘positive’ impacts (to be interpreted with caution)

Under extreme neglect of the environment, some children may develop emotional dullness or surface “emotional stability” (actually emotional isolation) too early, manifested as less crying and fussing. But this is a maladaptive defense mechanism that comes at the cost of poor emotional experiences and difficulties in interpersonal connections, rather than a healthy ability to regulate emotions.

- Perfunctorily neglected type

The influence of perfunctorily neglected type and directly neglected type is basically similar, but children who grow up in perfunctorily neglected type will be more patient. They will listen to some things and give some concise responses, but the content of the responses is usually emotionally indifferent and too brief.

### 5.4 The influence of directive style

1. Experiential guidance

- Negative impacts

- Limited autonomy and creativity: Solutions are mainly provided by parents, which may inhibit children’s autonomous decision-making ability and divergent thinking. Although Alice solved the problem of braids, she did not experience the process of actively thinking about “what else can I do besides cutting my hair?” The improvement of her self-efficacy in problem-solving mainly depends on the successful implementation of her parents’ plan.
- Dependent tendency: Children may habitually wait for their parents to “give answers” instead of actively thinking or trying to solve problems independently, which is not conducive to cultivating self-regulation learning ability.

B. Positive impacts:

- Safe base and positive parent–child relationship: Parents’ listening, investigation, and helping behaviors make children feel valued and supported, consolidate safe attachment, and promote harmonious and close parent–child relationships.
- Emotional stability and prosocial behavior: In a supportive environment, children’s emotions are more stable, they are more willing to listen to others, develop listening skills, and apply learned experiences to help peers.
- Internalization of rules and interpersonal harmony: Parental guidance often includes explanations of rules and others’ feelings, which helps children internalize social norms, behave more in line with situational requirements, and reduce conflicts.

1. Exploratory guidance

- Positive impacts (more comprehensive and avoids significant negative impacts)

On the basis of having all the positive effects of experiential guidance, exploratory guidance significantly promotes some other areas

- Advanced cognitive development: The process of jointly analyzing, generating options, and evaluating consequences effectively exercises analytical thinking, reasoning ability, metacognitive monitoring, and cognitive flexibility.
- Intrinsic motivation and self-efficacy: When the solution comes from the child’s active choice (even with guidance) and is successfully implemented (such as the success of Xiaohong’s case), the child experiences a strong sense of control, belief in “I can do it”, and self-efficacy, greatly enhancing intrinsic motivation.
- Creativity and adaptability: Encourage exploration of multiple possibilities and provide space for creative solutions. By considering the consequences of different options (such as changing seats which may avoid trouble but lose opportunities to make friends), children learn to weigh the pros and cons and adapt flexibly to situations (adaptive skills).
- Social understanding and problem-solving skills: In discussions, parents guide their children to consider the feelings and perspectives of others (such as “Why did your desk mate take your pen?” and “How might giving a pen make her feel?”), which directly promotes the development of perspective taking and empathy skills, as well as the formation of more mature conflict resolution strategies.
- Autonomy and sense of responsibility: Children participate in decision-making and executing plans, truly feel their influence on the process and results, and enhance their sense of autonomy and responsibility for their own choices.

## 6 Discussion and theoretical integration: building a key path for adaptive development

This study, through in-depth interviews and rigorous qualitative analysis, clearly depicts four typical styles of parents’ response to setbacks in kindergarten senior class children and their complex impact on children’s psychological and social development. The research results not only validate existing theories such as social learning theory, attachment theory, Erikson’s developmental stage theory, and Vygotsky’s ZPD theory, but also enrich our understanding of “effective support” in specific cultural contexts through data-rooted in the specific practices of parents from the Chinese mainland.

### 6.1 Reaffirmation and deepening of core discoveries

1. Negativity and neglect: high risk paths

This study, with detailed cases and in-depth analysis, strongly confirms the destructive effects of negative (especially accompanied by blame and corporal punishment) and neglectful parenting on early childhood development. They significantly increase the risk of internalizing problems (inferiority complex, anxiety, cowardice) and externalizing problems (aggression, blaming others) of children, seriously damaging their sense of security, self-worth, and quality of parent–child relationships. These effects are highly consistent with the formation mechanisms of learned helplessness models and insecure attachments. In the context of traditional Chinese culture, this kind of style may be related to the traditional concept of “strict education.” Some parents think strict criticism as a manifestation of “doing good for their children” (Zhou, 2004), but ignore the potential harm of this educational approach to children’s self-concept.

2. Guidance type: high adaptability path

The guidance-based approach, especially exploratory guidance, has been proven to be the optimal path for promoting adaptive development in kindergarten senior class children. It not only provides immediate emotional support and problem-solving, but more importantly, through scaffolding guidance, effectively cultivates children’s metacognitive ability, autonomous decision-making ability, social cognitive skills (empathy, perspective taking), and self-efficacy within their ZPD. This model perfectly combines the high responsiveness (accepting emotions, actively listening) and high demands (guiding thinking, encouraging experimentation, and taking responsibility) of authoritative parenting.

3. Indulgent type: contradictory sugar coating

The study reveals the contradiction of the indulgent style. It may bring high self-esteem, a sense of security, and a willingness to explore in the short term, but its core flaw – the lack of responsibility guidance and reality testing – lays hidden dangers for children’s future social adaptation (self-centeredness, low resilience). This is consistent with the research on the development paths of narcissistic traits and external attribution styles. Influenced by some traditional Chinese culture, some parents may cherish their first or youngest child very much (Wang and Chen, 2024), or have a strong preference for their son. So some parents may exhibit unconditional indulgence toward a particular child.

3. The importance of process quality

Research has found that whether parents are willing to listen and understand the facts, even if the final approach is not optimal, such as understanding first and then blaming/spoiling), is more likely to make children feel a certain degree of attention than completely refusing to listen or making conclusions directly, and may let children (to a limited extent) imitate the step of “understanding the situation”. However, only by combining knowledge of facts with constructive guidance (such as guidance type) can its positive value be maximized.

### 6.2 Theoretical inspiration

1. Integrating the ecological perspective of development

This study emphasizes that understanding parental behavior needs to be placed within a developmental ecology model. The coping style of parents is not only influenced by their personal traits such as personality and mental health, but also deeply rooted in their own growth experiences (intergenerational transmission), marital relationship quality, work pressure (microsystem), and broader social and cultural norms (macrosystem, such as different interpretations of “strength” and “obedience” in traditional Chinese culture). For example, a negative style may be related to certain cultures that emphasize “setback education” but misinterpret it as “suppression”; Neglecting type may be related to the fast pace of modern life and excessive parental pressure.

2. The core position of emotions

One of the core differences in all coping styles is how parents respond to their children’s emotions. The key to guiding success lies in its emotional socialization strategy: acknowledging emotions (“I know you’re upset/angry”), helping children label emotions, explaining their sources, and guiding constructive emotional expression and regulation strategies. However, the negative coping style (denying emotions), indulgent coping style (focusing only on emotions while ignoring behavioral consequences), and neglecting coping style (ignoring emotions) all have significant deficiencies in the process of emotional socialization. This confirms the importance of early experience in emotional intelligence development.

3. Exploratory guidance as best practice

This study strongly establishes “exploratory guidance” as the best practice for dealing with setbacks in kindergarten senior class children. It goes beyond simply imparting experience, but activates children’s active thinking through guided dialogue, open-ended questioning, and collaborative construction, cultivating their internal resources and psychological resilience to face future challenges. This provides vivid empirical support for Vygotsky’s zone of proximal development theory and social constructivist learning perspective.

### 6.3 Practical significance

1. Parental Education and Suppor

- It is urgent to carry out a special education project for parents of kindergarten senior classes, focusing on “coping with children’s setbacks”. The content should cover: understanding the psychological development characteristics of 5–6 year old children (especially emotions and sociality), identifying parents’s own coping styles and their potential impacts, learning the specific steps of “exploratory guidance” listening,empathy, understanding, collaborative analysis, exploratory options, supportive selection, retrospective summary managing parents’s own emotions (avoiding projecting anxiety or anger onto the child). At the same time, experts should convey to parents the scientific and rational approach to traditional Chinese culture, in order to avoid some parents blindly believing in erroneous traditional concepts that may lead to negative educational methods (Ding and Liu, 2021).
- Promote the concept and techniques of “emotional education” to help parents become “containers” and “guides” for their children’s emotions.
- Provide psychological support and resource links for parents who are under excessive pressure or have difficulties (such as depression) to reduce the occurrence of neglectful or inappropriate negative behaviors.

2. Co–education between kindergarten and home

- Kindergartens should incorporate “setback education” into their curriculum system, using forms such as picture books, role-playing, and social drama to help children learn skills in recognizing emotions, expressing needs, and resolving conflicts.
- Teachers should keenly observe children’s performance and traces of family influence when Children encounter setbacks in their daily work, establish positive communication channels with parents, share observations, and timely convey scientific parenting concepts and successful cases of “exploratory guidance” on the basis of respecting parents. Teachers should also persuade parents to treat traditional Chinese culture in a right way—take the essence and discard its dross (Wang and Ouyang, 2011), so as to reduce the negative impact of bad elements in traditional culture on parents’ education methods.
- Establish parent support groups or workshops to promote learning and exchange of experiences in coping with setbacks among parents.

### 6.4 Social advocacy

Media and social organizations should advocate a scientific and rational parenting philosophy, abandon outdated concepts of corporal punishment and punitive education, and be wary of new types of parenting problems that are unprincipled and overly protective. They should promote a positive parenting culture centered on “exploratory guidance.”

### 6.5 Research limitations and future development directions

Firstly, the research data comes from parents’ interviews, which may be influenced by social expectations. Future research can combine observational methods or Children’s interviews to obtain multi-source data. Secondly, the study adopts a cross-sectional design, which may hard toreveal the causal direction between parental coping styles and child development. In the future, longitudinal tracking studies can be conducted to explore the dynamic interaction between the two. Thirdly, the researcher’s own background as an educator may affect data interpretation, and although measures such as reflective recording have been taken, researcher’s bias cannot be completely eliminated. Future research can invite researchers from diverse backgrounds to participate together, enhancing the objectivity of the research.

## 7 Conclusion

This grounded research, based on in-depth interviews and qualitative analysis, analyzes four typical styles of parents of kindergarten children in Chinese Mainland in dealing with children’s setbacks and their profound impact on children’s psychosocial development. This study combines rich raw materials and educational psychology for analysis, revealing that kindergarten senior classes are a critical shaping period for children to shape their self-awareness, learn emotional management, develop social skills, and construct ways of coping with the world. The way parents deal with their children’s setbacks at this stage is like a carving knife, deeply shaping the trajectory of their children’s psychological development. Abandoning the harm of negation and neglect, transcending the short-sighted indulgence, and embracing exploratory guidance centered on respect, understanding, and cooperation are the most solid foundations that parents can lay for their children’s future psychological resilience and happy life. With the development of the times, the connotation and practice of exploratory guidance will continue to enrich and evolve on the basis of drawing scientific evidence (such as positive psychology and neuroscience’s new discoveries on brain plasticity) and respecting children’s subjectivity. This study calls on parents, educators, and all sectors of society to work together to provide every growing child with an “exploration boat” that can help them learn to steer themselves.

## Data Availability

The original contributions presented in the study are included in the article/supplementary material, further inquiries can be directed to the corresponding author.
